# Monomerization of the photoconvertible fluorescent protein SAASoti by rational mutagenesis of single amino acids

**DOI:** 10.1038/s41598-018-33250-z

**Published:** 2018-10-19

**Authors:** Ilya D. Solovyev, Alexandra V. Gavshina, Aditya S. Katti, Alexey I. Chizhik, Leonid M. Vinokurov, Grigory D. Lapshin, Tatiana V. Ivashina, Maria G. Khrenova, Igor I. Kireev, Ingo Gregor, Jörg Enderlein, Alexander P. Savitsky

**Affiliations:** 1 A.N. Bach Institute of Biochemistry, Research Center of Biotechnology of the Russian Academy of Sciences, Moscow, Russia; 2 M.V. Lomonosov Moscow State University, Department of Chemistry, Moscow, Russia; 3 University of Göttingen, Third Institute of Physics – Biophysics, Friedrich-Hund-Platz 1, 37077 Göttingen, Germany; 4 Branch of Shemyakin and Ovchinnikov Institute of Bioorganic Chemistry, Russian Academy of Sciences, Pushchino, Moscow Region Russia; 5 Skryabin Institute of Biochemistry and Physiology of Microorganisms, Russian Academy of Sciences, Pushchino, Moscow Region Russia; 6 A.N. Belozersky Institute of Physico-Chemical Biology, M.V. Lomonosov Moscow State University, Moscow, Russia

## Abstract

Photoconvertible fluorescent proteins (PCFPs) are widely used as markers for the visualization of intracellular processes and for sub-diffraction single-molecule localization microscopy. Although wild type of a new photoconvertible fluorescent protein SAASoti tends to aggregate, we succeeded, via rational mutagenesis, to obtain variants that formed either tetramers or monomers. We compare two approaches: one is based on the structural similarity between SAASoti and Kaede, which helped us to identify a single point mutation (V127T) at the protein’s hydrophobic interface that leads to monomerization. The other is based on a chemical modification of amino groups of SAASoti with succinic anhydride, which converts the protein aggregates into monomers. Mass-spectrometric analysis helped us to identify that the modification of a single ε-amino group of lysine K145 in the strongly charged interface AB was sufficient to convert the protein into its tetrameric form. Furthermore, site-directed mutagenesis was used to generate mutants that proved to be either monomeric or tetrameric, both capable of rapid green-to-red photoconversion. This allows SAASoti to be used as a photoconvertible fluorescent marker for *in vivo* cell studies.

## Introduction

Fluorescent proteins are widely used for the specific fluorescent labeling of cells, organelles, and individual proteins^1,2^. Several methods of sub-diffraction localization microscopy (PALM^3,4^ and fluorescence correlation microscopy (FCS^5^ use photoconvertible GFP-like proteins. The first protein of this type was Kaede, obtained from a scleractinian (stony) coral^6^. After expression and maturation, this protein shows initially fluorescence in the green spectral region. However, irradiation with blue light modifies its fluorescence excitation and emission spectra and converts its emission from green to the red. The green fluorescence of Kaede stems from a classic GFP chromophore, formed from a His66-Tyr67-Gly68 tripeptide by an autocatalytic reaction. Irradiation with blue light induces cleavage of the Nα–Cα bond in His66 via a beta elimination reaction. Subsequent proton loss at His66-Cα results in the formation of a double bond between His66-Cα and –Cβ, leading to the expansion of the conjugated π-system to include the imidazole ring of His62^7^. Further research identified other fluorescent proteins capable of similar chromophore photoconversion and with similar fluorescent properties: EosFP^8^, found in the stony coral *L. hemprichii*, Dendra from the soft coral *Dendronephthya sp*., and KikG found in the stony coral *F. favus*. More recently, protein engineering of a cyan-emitting FP (cFP484) from the coral *Clavularia sp*. led to the design of a green-to-red photoconvertible CFP, mClavGR2^9^, and its improved variant mMaple^10^.

Wild type fluorescent proteins obtained from corals are prone to aggregation. Expression of such proteins in cells results in the formation of fluorescent granules^11^. Some of these proteins form oligomers, specifically homodimers^12^ or homotetramers^11–20^. It is thought that large aggregates can block intracellular pores, including the nuclear pore^21,22^. In order to minimize this effect, fluorescent proteins are usually modified to be monomeric. It is typically accomplished by directed evolution^23^. A monomeric FP conformation is preferable for sub-diffraction single-molecule localization microscopy, since the precise position of the fluorophore is determined by fitting the center position of its image (point spread function), and a larger label size can thus affect the localization accuracy^24^. However, stable tetramers can be useful due to their enhanced absorbance, which maybe important e.g. when using the protein as acceptor in a FRET pair^25^ or to increase their overall brightness^11^.

Study of the structure of GFP-like proteins and their oligomers has shown that di- and tetramers of these proteins are arranged in a similar four-can pack^26^ pattern. The tetramer structure was first found for the protein DsRed^14,16^. Two types of interfaces were found in these tetramers: the first one is typical for many high-affinity protein–protein interaction surfaces (AC interface) and consists of hydrophobic residues, surrounded by polar residues^27^; the second consists almost exclusively of polar and charged residues (AB interface)^14^. The tetramer subunits are interfaced in a symmetrical manner. Each interface is formed by contacts between the same amino acid sequences of two subunits. The overall structure of the tetramer looks like a dimer formed by dimers^16^. These properties suggest a strategy for inducing monomerization of a protein by using rational mutagenesis, where the amino acids in the interface region are prime targets^8,28,29^. For instance, in DsRed, the introduction of a single mutation into the charged residue interface results in the formation of a dimer. However, the mutant protein exhibits weak fluorescence and takes longer time to mature than the wild type protein. However, fluorescent properties can be restored by four subsequent iterative cycles of evolution^28^. In order to obtain brightly fluorescent monomers, it is usually necessary to introduce mutations into both sides of the inter-subunit interfaces of the tetramer^8,28,29^. The first monomeric red fluorescent protein was obtained by introducing 33 mutations, 13 of them were located in the tetramer interfaces^28^. The number of mutations required for monomerization can vary, depending on the protein. For example, the creators of the photoconvertible mEOS protein managed to obtain a monomeric protein with only two mutations, one in each tetramer interface^8^.

We have recently purified a novel GFP-like protein, SAASoti, from the coral *Stylocoeniella armata*. We demonstrated that it is photoconvertible from green to red fluorescence under 405 nm illuminations^30^. Under 470 nm illumination green fluorescence of SAASoti and its variant are reversible photobleached without photoconversion to the red form. The phenomenon can be explained by chromophore protonation that was confirmed by increase in absorption at 400 nm (chromophore protonated form) and decrease at 509 nm (anionic form). This photo-induced photoswitching can be repeated several times with the same sample without previous fluorescence intensity loss^31^. The aim of the work presented here is to determine the oligomeric state of this protein, to obtain a monomeric variant and to improve its fluorescent properties to make it useful for single-molecule localization microscopy.

## Results

### Oligomer size and interface structures in oligomers

**Figure 1:**
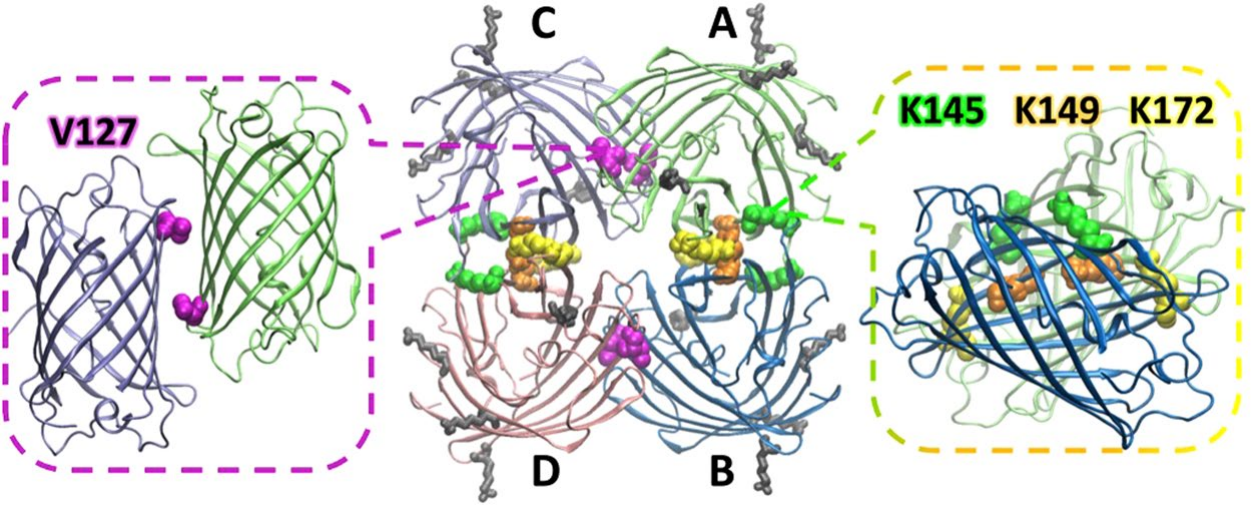
SAASoti tetrameric model structure (based on the Kaede structure PDB code: 2GW3). Right: rotated charged AB interface, K145, K149 and K172 are shown in green, orange and yellow, respectively; center: 3D model with two distinct interfaces – AB and AC; left: rotated hydrophobic AC interface, V127 is shown in magenta. Surface-facing lysines K39, K41, K112 and K139 are shown in grey.

3D tetrameric model of SAASoti (Fig. 1) was constructed based on the known structure of Kaede^32^ (PDB ID: 2GW3). Sequence alignment of SAASoti with Kaede and avGFP (wild type GFP) is described in^30^. The relevant residues in the Kaede structure were replaced by those from the SAASoti sequence with the help of the Mutate Residue module of the VMD program package^33,34^.

**Figure 2:**
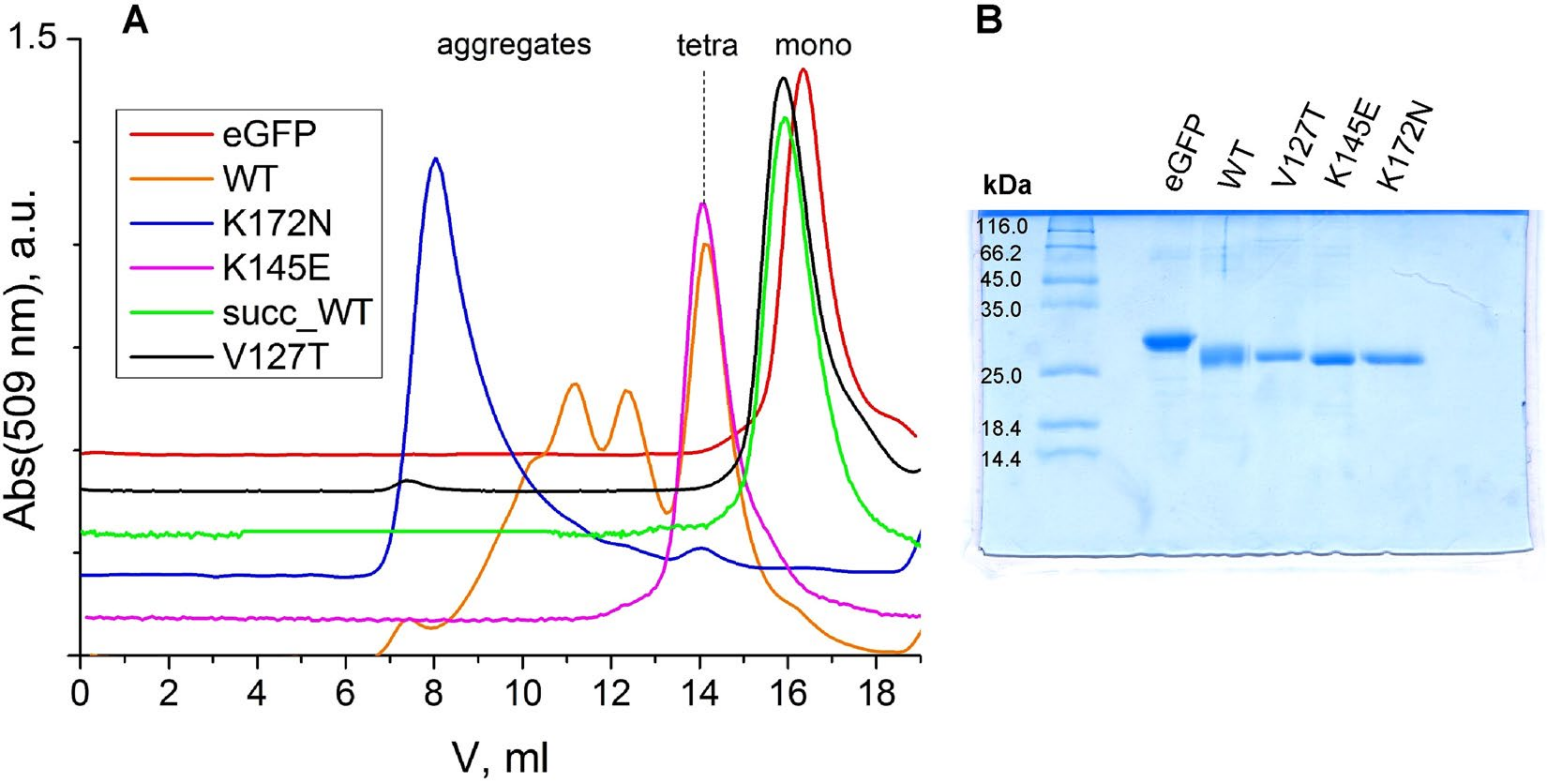
(**A**) Size-exclusion chromatography of different SAASoti mutants, succinylated SAASoti and eGFP (monomer control). Superdex G200 100/20 GL column, buffer Tris-HCl 100 mM, CaCl2 10 mM, pH 7.6, detection by absorption at 509 nm; (**B**) PageBlue stained 4–15% SDS-PAGE after all protein purification procedures. Lanes: kDa- SM0431 Fermentas calibration kit; 1 – eGFP; 2 – wild type SAASoti; 3 – V127T SAASoti; 4 – K145E SAASoti; 5 – K172N SAASoti.

Photoconvertible SAASoti protein was expressed in *E. coli* and purified. Size-exclusion chromatography data (Fig. 2) show that SAASoti exists in aggregated form and as a tetramer (preferably). Structure analysis of SAASoti model revealed two surface-facing cysteine residues – C21 and C117. Interestingly, that wild type SAASoti still forms stable oligomers under reductive conditions in the presence of DTT, as only 7.36 ml peak disappears (Supplementary Table S1). Structural analysis of the SAASoti model indicated that the interfaces between the tetramer subunits contain lysines. Thus we decided to determine the role of these amino acid residues in the oligomerization by succinilating SAASoti amino groups with succinic anhydride, because that should mask the charge of these groups and disrupt electrostatic interaction. Treatment of SAASoti samples with succinic anhydride at a molar ratio of 1:20 led to the disappearance of octa- (247 kDa) and tetra- (104 kDa) aggregates and resulted in the appearance of peaks with masses that can be attributed to the monomeric form (22 kDa) of the protein according to size-exclusion chromatography experiments (Fig. 2).

For determining which specific ε-amino group modification caused protein monomerization, the fraction corresponding to the 22 kDa peak (Fig. 2) was analyzed using mass spectrometry. The tryptic peptides were analyzed using a MALDI-TOF/TOF mass spectrometer. Trypsin cleaves the peptide chain after lysine and arginine residues and full MS-spectra of peptides before and after succinilation presented on Supplementary Fig. S1. If a lysine residue is modified, its side chain amino group will be replaced with a carboxyl group. Trypsin cannot cleave a polypeptide at such a site^35^. If a lysine residue is modified by succinic anhydride at its ε-amino group, its molecular mass increases by 100.7 Da (Supplementary Fig. S2).

29 mass peaks were identified by MALDI in the resulting tryptic peptide mixture. The identified peptides encompass 47% of the SAASoti sequence. However, lysines in the SAASoti sequence are not evenly distributed and 12 of the 17 lysines were located in the tryptic peptides as identified by MALDI. Trypsin cleaves peptide bond at the carboxyl side of the lysine residues K34, K84, K184 and these residues were not exposed to succinyl modification. In other words, 15 of the 17 lysine residues were identified as modified or not (88% lysine coverage, Supplementary Fig. S3). Among this set of peaks, we identified several that had a mass increase of 100.7 Da as compared to the calculated peptide mass. This increase is caused by the modification of the amino groups by succinic anhydride.

We analyzed all tryptic peptides with molecular masses corresponding to one or two modified lysines (Table 1). According to the constructed 3D model, the lysine residues 145, 149 and 172 are located in the AB interface of the tetramer (Fig. 1). Their modification can affect the oligomerization state of the protein. As it can be seen from the increased masses of the corresponding peptides, lysine residues 36, 41, 112 and 139 are also modified in the monomer fraction. However, the model predicts that these residues are located on the surface of the tetramer (Fig. 1).

**Table 1:** Amino acid sequences of modified tryptic peptides identified by MALDI-TOF/TOF. Masses are given in Daltons.

From-to	Tryptic peptide sequence	Mass	with 1 succinic	with 2 succinic	with 3 succinic
35–45	A**K**PYEG**K**QNLK	1275.7		1475.7	
85–95	QSFPEGYSWER	1385.6			
96–112	TFAFEDGGFCTASADIK	1779.8			
96–114	TFAFEDGGFCTASADI**K**LK	2021.0	2120.9		
139–145	**K**TIQWEK	932.5	1032.5		
139–149	**K**TIQWE**K**SIEK	1389.8	1489.8	1589.9	
140–149	TIQWE**K**SIEK	1261.7	1361.7		
146–158	SIE**K**MTVSDGIVK	1406.8	1506.8		
139–158	**K**TIQWE**K**SIE**K**MTVSDGIVK	2320.3		2520.3	2620.3
140–158	TIQWE**K**SIE**K**MTVSDGIVK	2192.2		2392.2	
159–172	GDITMFLLLEGGGK	1450.8			
159–174	GDITMFLLLEGGG**K**YR	1770.0	1869.9		
175–182	CQFHTSYK	1013.5			
185–201	**K**VVEWPQSHYVEHSIER	2068.0	2168.0		
186–201	VVEWPQSHYVEHSIER	1940.0			
202–218	TNDDGTQFELNEHAVAR	1916.9			
202–222	TNDDGTQFELNEHAVARLNEI	2386.1			

### Site directed mutagenesis

Site directed mutagenesis was used to disrupt the strongly charged interface between the tetramer subunits. Isomorphic amino acid residues were chosen for substitution so as not to disrupt the protein folding or the inner surroundings of the chromophore. The following mutations were introduced: K145N, K145E, K149E, K149N and K172N. Among these mutant proteins only one, K172N, was prone to aggregation, while the others were shown to be tetrameric as shown by size-exclusion chromatography (Fig. 2) and 2fFCS (see below).

**Figure 3:**
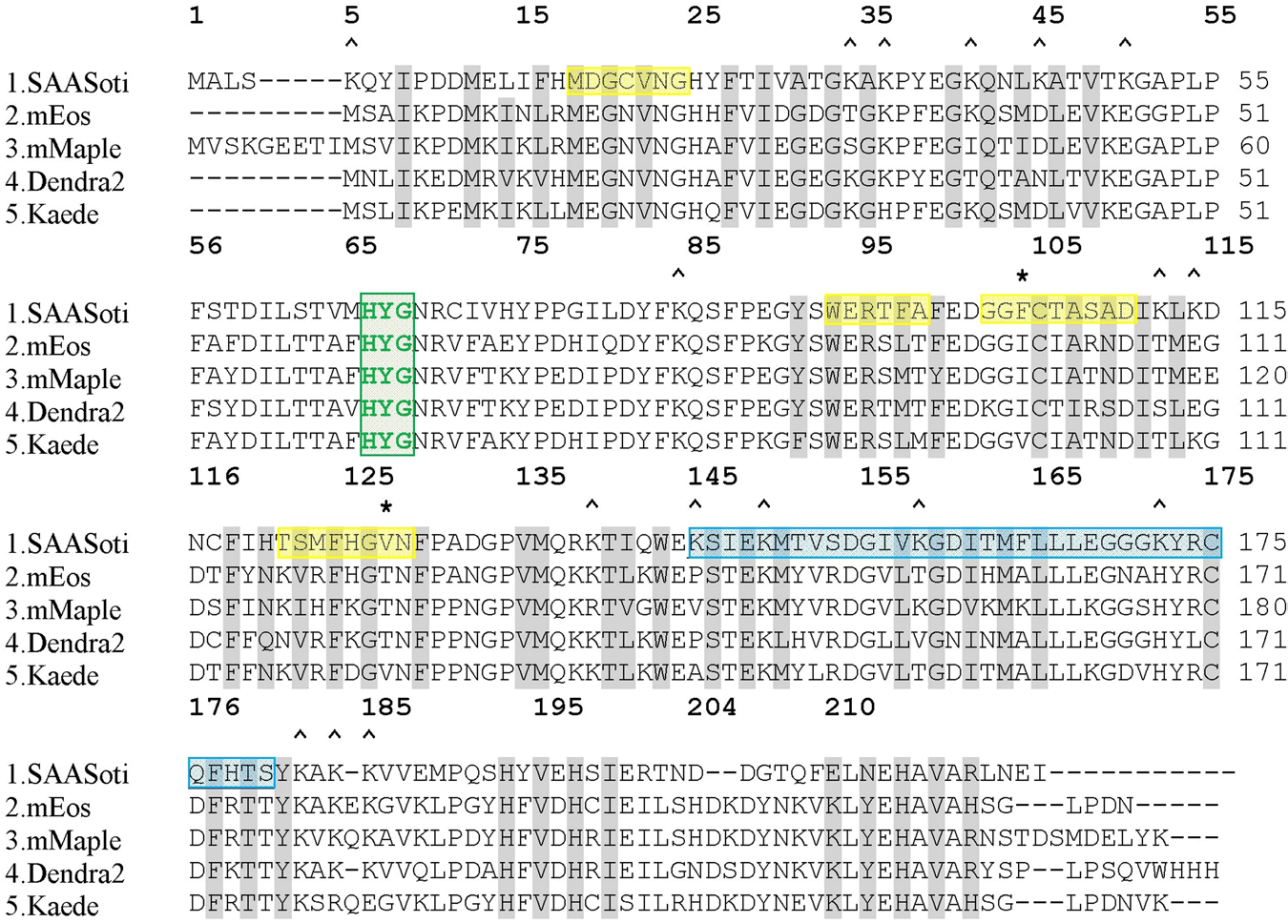
Sequence alignment of different photoconvertible FPs. Chromophore forming amino acids are shown in green; hydrophobic AC interface is highlighted in yellow; charged AB interface is highlighted in blue; ^ – marks all lysine residues; amino acid residues facing into the β-barrel are highlighted in grey. Amino acid residues substituted by site-directed mutagenesis – V127, K145, K149 and K172 – are shown in red. 1, 5 etc. – SAASoti amino acid numbering.

Based on our preliminary data showing that the putative charged interface cannot be disrupted by amino acid substitutions, the hydrophobic interface was chosen for further modification. Sequence and structure alignment with the previously successfully monomerized FPs Dendra2, Kaede, DsRed, mEos revealed two amino acid residues – V127 and F104 – the modification of which could potentially induce SAASoti monomerization (Fig. 3). We have chosen V127T mutation because threonine is likely to disrupt the hydrophobic interface via their bulky hydrophilic side chains. Based on size-exclusion chromatography only this mutant have retention time similar to monomeric eGFP up to mM concentrations. V127T SAASoti partial dimerization at high concentrations (0.22 mM) is most likely caused by S-S bond formation between the subunits. After DTT was removed from eluted fractions by dialyzing against 20 mM Tris-HCl pH 7.4, 150 mM NaCl and V127T SAASoti was concentrated we obtained 0.1 mM fraction. Repeated gel-filtration chromatography at non-reductive conditions revealed monomeric state of the protein (Supplementary Fig. S4**)**.

**Figure 4:**
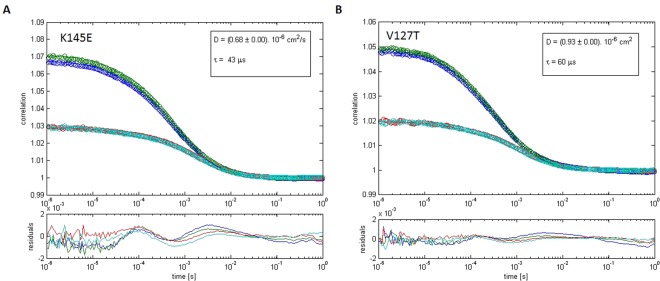
2fFCS measurements of fluorescent proteins diffusion. FP’s curves were fitted by a triplet model. Dots – data points, lines – fitting curves. Green and cyan curves are first and second focus autocorrelation functions, red and blue curves – cross-correlation curves between two focal volumes. A – K145E SAASoti, B — V127T SAASoti.

Two-Focus Fluorescence Correlation Spectroscopy (2fFCS) is one of the most sensitive methods for studying protein diffusion^36^. Based on a modified fluorescence correlation spectroscopy (FCS)-setup, this method is improved by introducing an external ruler for measuring the diffusion time by generating two laterally shifted and overlapping laser foci at a fixed and known distance. Calculation of the diffusion coefficient is done by analyzing the measured 2fFCS autocorrelation function *G*(*t*, *δ*) using triple state model. Using 2fFCS, we calculated diffusion coefficients of *D* = 68 ± 1 µm^2^/s for SAASoti (K145E) and 72 ± 1 µm^2^/s for wild type SAASoti. The data obtained is consistent with the diffusion coefficient of a tetramer^37^ or an even larger oligomer (Supplementary Fig. S5). The diffusion coefficient for V127T SAASoti was estimated to be equal to *D* = 93 ± 1 µm^2^/s (Fig. 4) and is little bit less than measured value 117 ± 2 µm^2^/s and previously published 97 µm^2^/s for eGFP measured by FCS^37^. Thus, SAASoti (V127T) is supposed to be in a monomeric state. 2fFCS analysis of the oligomeric states of the mutants showed that the V127T mutation caused SAASoti to remain a monomeric state.

### Physicochemical properties of different SAASoti mutants

A next step of our study was concerned with further physicochemical characterization of the obtained mutants and the comparison of their properties to those of fluorescent photoconvertible protein Dendra2. Absorbance and fluorescence properties of the GFP-type chromophores depend on their tyrosine (Y67) protonation state. Only the anionic form of the GFP type chromophore is fluorescent^38^. Extinction coefficients were calculated as the ratio between the absorbance of the anionic form of the SAASoti chromophore and the calculated absorbance at 280 nm of the highly purified protein (SDS PAGE >90%) as described in^39^.

In the case of K145E SAASoti, ε (509 nm, green form) was equal to 66000 М^−1^cm^−1^ while this value was as large as 75000 М^−1^cm^−1^ for V127T mutant. Determination of ε for the red form (579 nm,) was complicated due to photobleaching during protein photoconversion, and was estimated to be 24000 М^−1^cm^−1^ at pH 7.5 for both mutants. For Dendra2, these coefficients are equal to 45000 М^−1^cm^−1^ and 35000 М^−1^cm^−1^, respectively. SAASoti has noticeably higher extinction coefficients in its green form. The quantum yield (Φ) of SAASoti was determined at pH 7.5 for the green and red forms using a nanocavity-based fluorescence quantum yield measurement (described below). Results are presented in Table 3.

**Figure 5:**
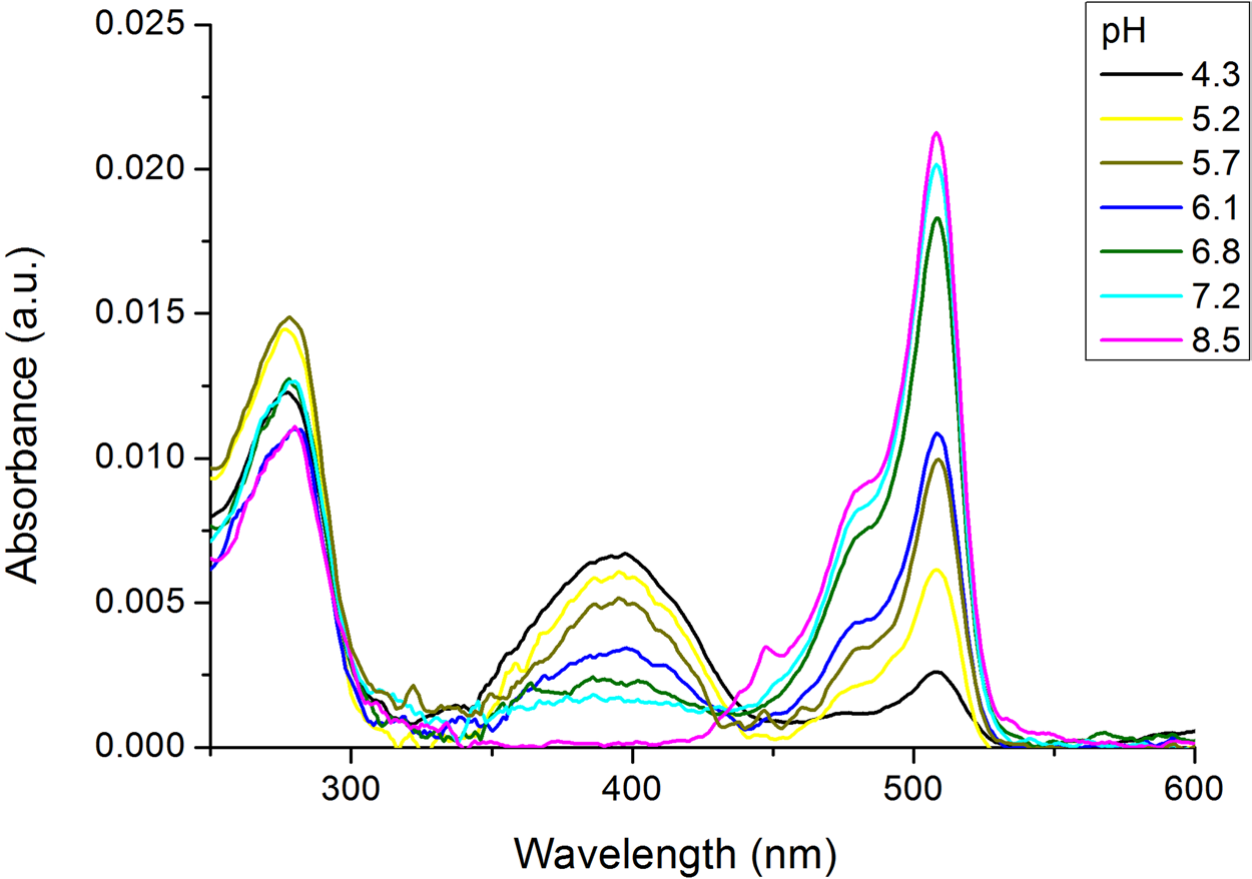
Absorption spectra of the V127T variant of SAASoti in 200 mM sodium phosphate buffers with varying pH.

**Figure 6:**
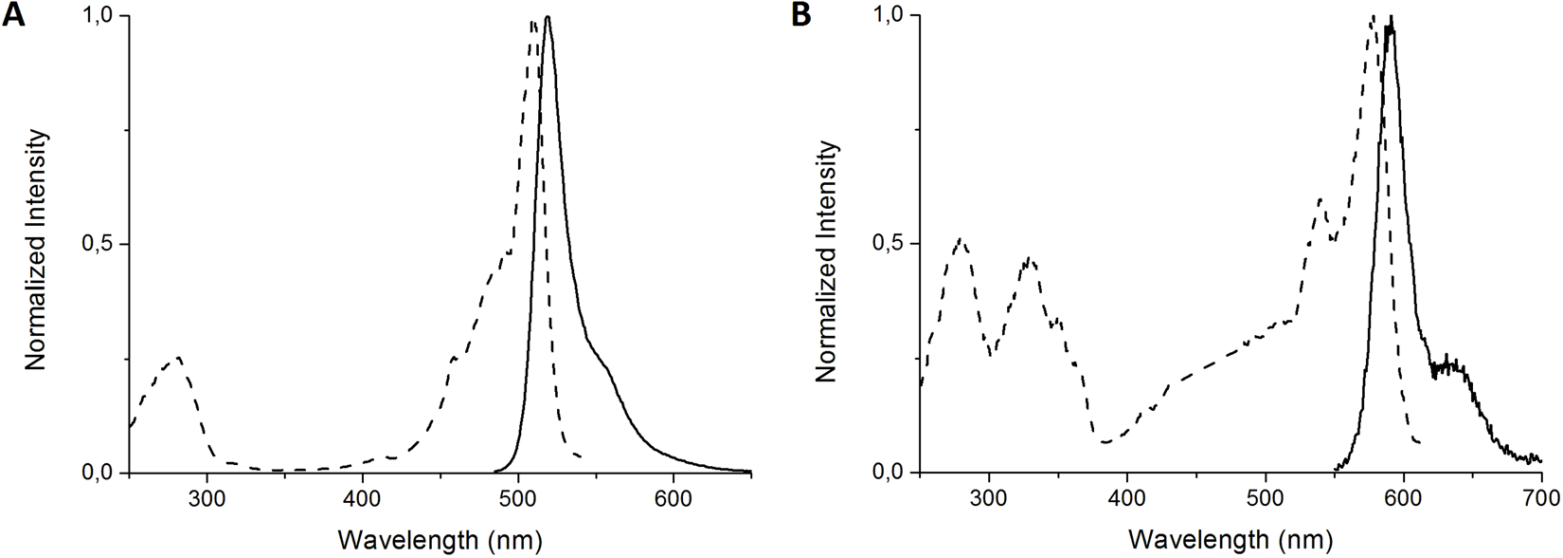
Normalized excitation and emission spectra of the K145E mutant before photoconversion (**A**, green state) and after complete photoconversion (**B**, red state).

The green anionic form has an absorption and excitation maximum at 509 nm (Figs 5 and 6a), while the red form has a maximum of excitation at 579 nm (Fig. 6b). Only anionic form of both green and red chromophores is fluorescent. Correspondently, two pH equilibria that control by pK_1_ and pK_2_ and characterize chromophore anion to the neutral state transition were calculated from dependences of fluorescence intensities for green and red forms, respectively.

As it can be seen from Table 4, modification of the hydrophobic interface with uncharged amino acid residues (V127T) does not result in a significant change of the pK_1_ and pK_2_ as compared to wt SAASoti, while substitutions in the charged interface increase the difference between the green and red forms. Comparing V127T SAASoti to Dendra2 shows more acidic pK values in the case of SAASoti, thus the fluorescent form of SAASoti will be prevalent in the cytoplasm. Changes in the amino acid content of the surface and changes in the oligomerization cause a local pH change in the protein, thus altering the pK of the chromophore.

### Green-to-red photoconversion of SAASoti mutants in cuvette

**Figure 7:**
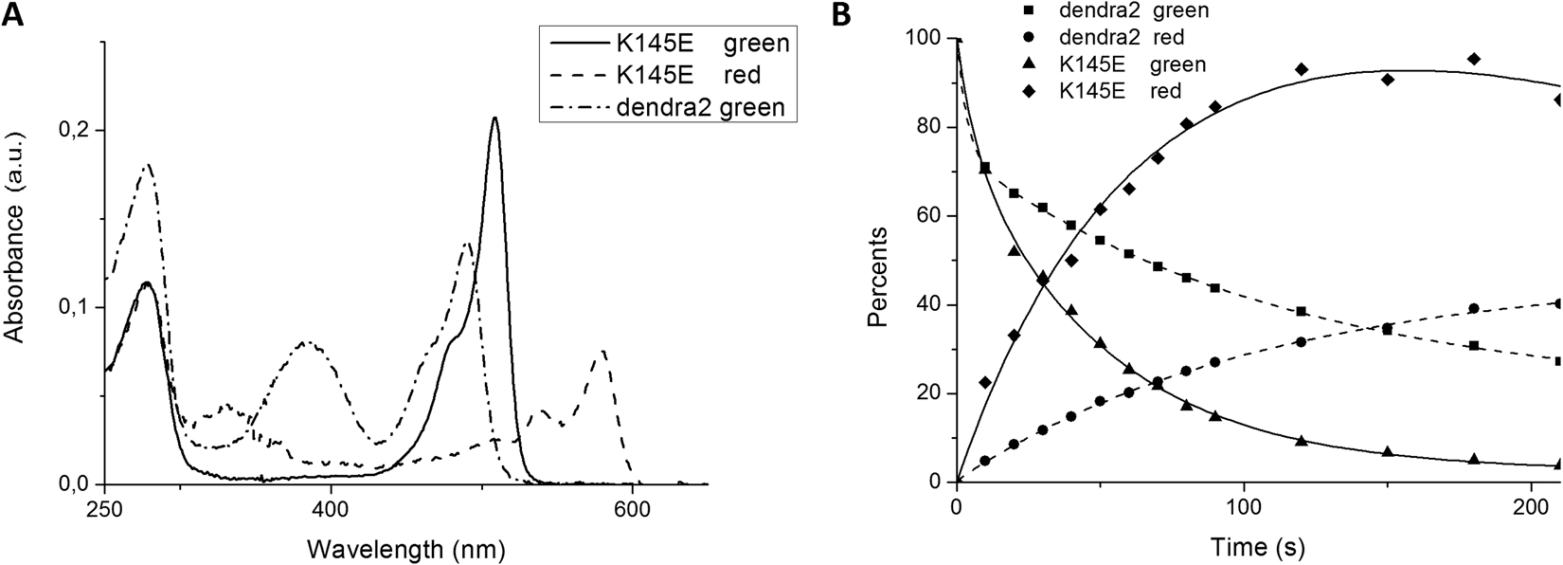
(**A**) Absorbance spectra of K145E (green state), K145E (red state) and Dendra2 (green state) in Tris-HCl pH 7.5. (**B**) Kinetics of the green-to-red photoconversion of the fluorescence of the K145E mutant and Dendra2 upon irradiation with a 405 nm laser. (Dendra2 green excitation (ex.) 465 nm, emission (em.) 507 nm, red ex. 530 nm, em. 573 nm, K145E green ex. 475 nm em. 519 nm, red ex. 550 nm, em. 590 nm all with 5 nm slits).

The excitation and emission spectra of the green form of K145E suggested that the chromophore is in an anionic form (Fig. 6A), similar to mEos. Unlike mMaple and Dendra2, K145E does not show a noticeable presence of the neutral form of the chromophore within the 380–400 nm region and it shows only a very weak absorbance at 405 nm (Fig. 7A). Only the protonated form (λ_abs_ = 385 nm, Figs 5 and 7A) can be irreversibly photoconverted into the red form. Nevertheless the photoconversion rate for K145E was much larger than that for Dendra2 (Fig. 7B). In order to study the impact of pH on SAASoti photoconversion, we induced photoconversion in its neutral state using 405 nm laser light with 30 mW intensity. As for the wild type SAASoti, all SAASoti mutants remained photoconvertible between their green and red fluorescent forms. The fluorescence maxima and shapes of both emission peaks for all mutants are nearly identical to those of the wild type protein. However, certain photochemical properties of the mutant proteins differ from those of the wild type (Table 4).

**Figure 8:**
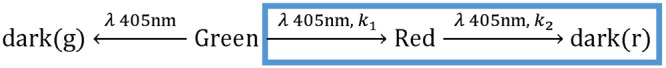
Kinetic model of SAASoti photoconversions.

The photochemical reaction of conversion from green to red involves a non-reversible cleavage of the polypeptide chain. Under constant light intensity at fixed wavelength 400 nm, this reaction can be formally described as a sequential monomolecular reactions (Fig. 8). According to Fig. 7, the red form of the protein is the most easily observed, as it can be quantified by monitoring the fluorescence at 590 nm (in the case of SAASoti) or 580 nm (in the case of Dendra2). Since a change in pH alters the amount of the neutral photoconvertible form, green to red photoconversion constant *k*_1_ and red form photobleaching constant *k*_2_ represent only apparent rate constants.

**Figure 9:**
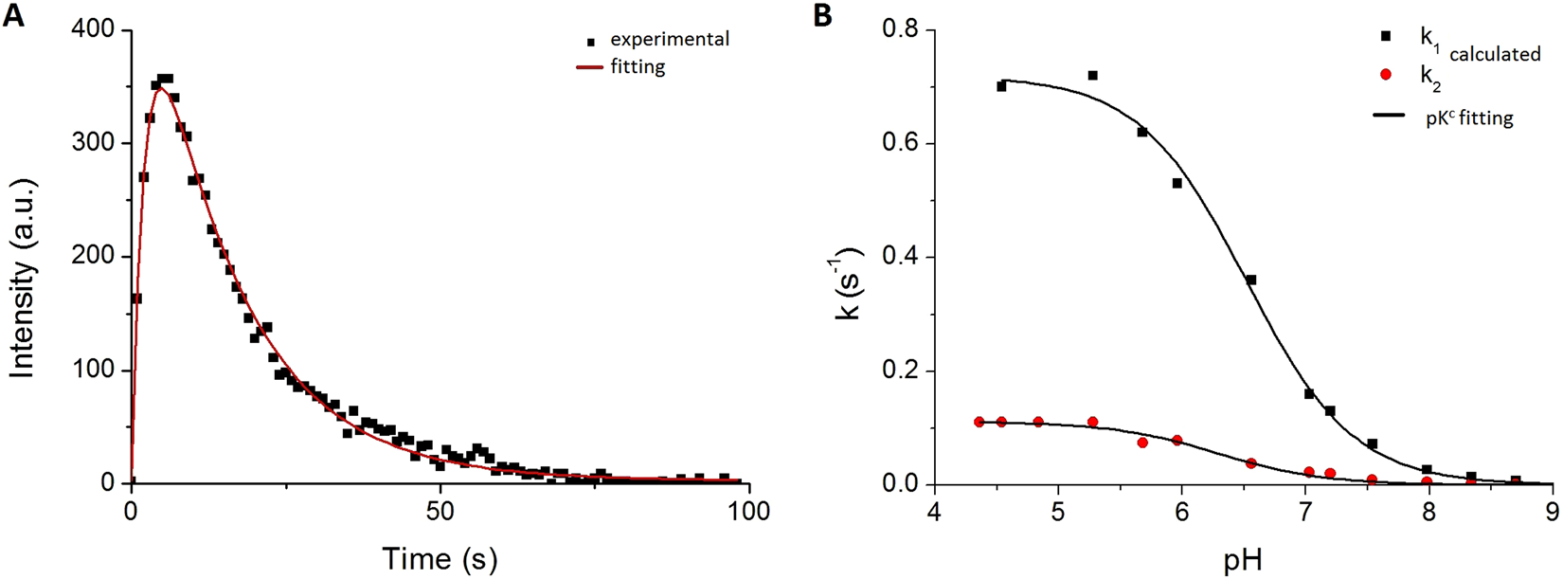
Analysis of SAASoti photoconversions. (**A**) Kinetic of SAASoti V127T photoconversion at 395 nm excitation and 580 nm emission at fixed pH. (**B**) pH profile of the rate constant of green-red photoconversion (*k*_1_), and rate constant for red form photobleaching (*k*_2_). Points are the experimental data, lines are fittings.

Thus, the maximal rate constants are the key parameters describing FP photoconversion (Fig. 9). For studied SAASoti mutants, the values of rate constants are in the range 0.6–0.9 s^−1^ for *k*_1_ and 0.07–0.11 s^−1^ for *k*_2_ (Table 4). For Dendra2, one finds *k*_1_ = 0.03 s^−1^ with no observable photobleaching. pK_1_^c^ and pK_2_^c^ that control photoconversion of green and red forms and calculated from the pH profile of *k*_1_ *and k*_2_ (Fig. 9) practically for all mutants are not coincide with pK_1_ and pK_2_ that control anion to neutral equilibrium of chromophore for both green and red forms (Table 4).

Comparison of values obtained for SAASoti and Dendra2 indicate that the photoconversion of SAASoti proceeds by more than an order of magnitude faster than for Dendra2. This allows for using much lower dose of 400 nm light for protein photoconversion, e.g. in cell models expressing SAASoti.

### Green-to-red photoconversion of V127T-SAASoti versus Dendra 2 in living cells

In the case of the cell microscopy V127T-SAASoti converts into the red form much faster than Dendra2 but not so fast as in cuvette. Constant of V127T-SAASoti conversion is equal 0.33 s^−1^ versus 0.013 s^−1^ for Dendra2 (Table 4). SAASoti’s time dependence includes fast conversion and then fast bleaching. Bleaching at 560 nm of V127T-SAASoti consist of two main exponents 0.4 s^−1^ and 0.04 s^−1^, while Dendra2 has a 0.28 s^-1^ and a 0.0015 s^−1^ component. Fast photobleaching is critical for PALM protocol.

### Expression in mammalian cells and sub-diffraction microscopy

**Figure 10:**
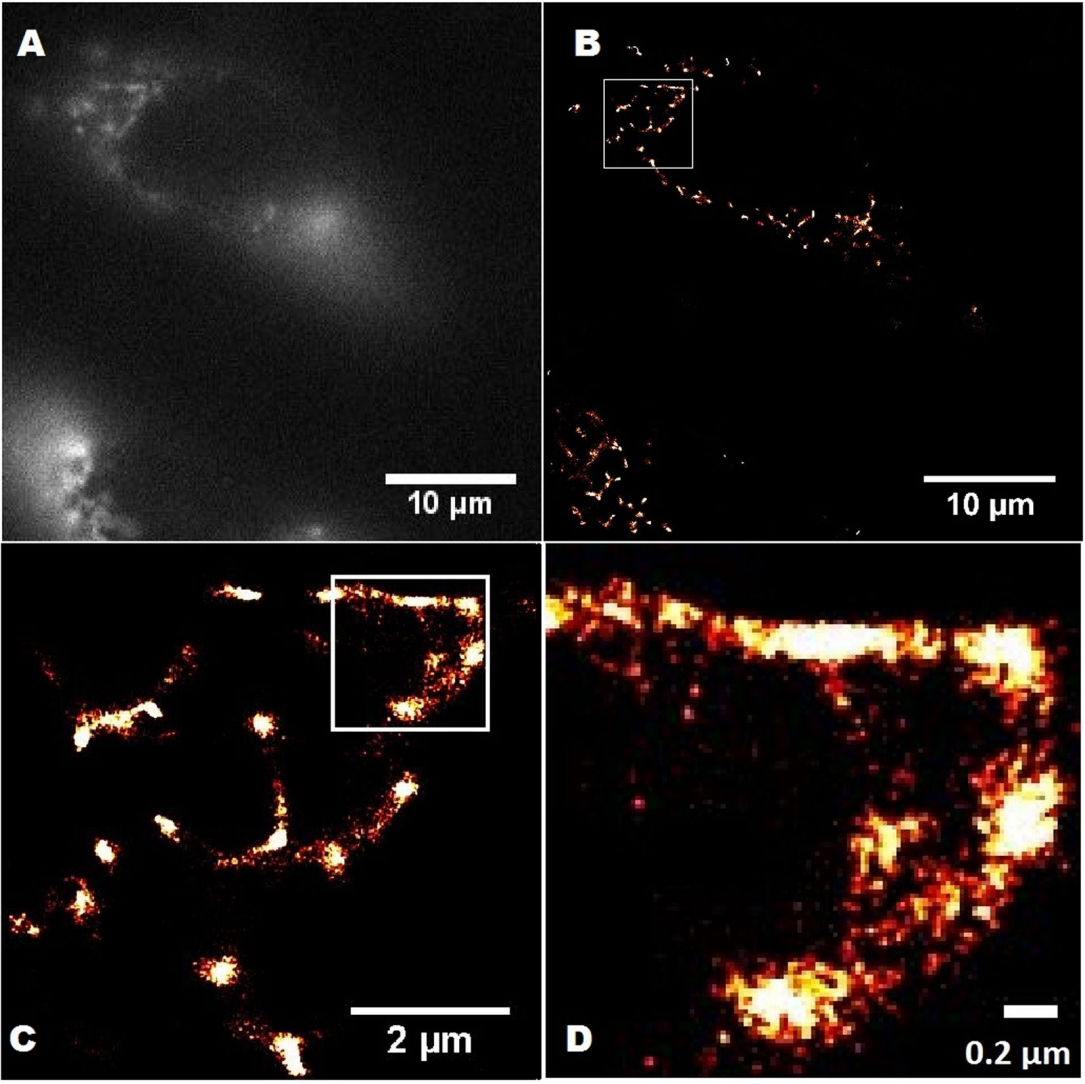
Near-TIRF widefield image (**A**) and PALM different scales images (**B**–**D**) with region of interest of Hep2 cells transfected with pVimentin-SAASoti/V127T vector. Images were captured using a TIRF 100x objective, using a CCD camera with 160 nm effective pixel size. Exposure time per frame was 30 ms. In near-TIRF mode (with slight sample penetration) a 561 nm laser was used for excitation, with a 580 nm emission bandpass filter in the detection pathway. A 405 nm laser was used for photoconversion from green to red. One measurement cycle involved photoconversion with a 405 nm laser for 1 frame and then excitation with a 561 nm laser for 3 frames (1 – registration of the signal at 561 nm, 2,3 – bleaching of the signal at 561 nm). The whole measurement comprised of 500 cycles. Every second frame of the cycle was used for analysis. Images were analyzed using the ThunderSTORM plugin of ImageJ.

Oligomerization and aggregation of fluorescent fusion proteins often prevents from obtaining reliable data on cellular and subcellular structures. Moreover, such multimeric proteins and protein aggregates are often toxic to cells^11^. Another problem is the preferred localization of these fusion proteins into specific organelles due to the structural properties of the fluorescent protein. Thus, in the absence of special intracellular localization signals, the preferred localization for fluorescent proteins is the cytoplasm. The obtained monomeric V127T protein displays cytoplasmic localization in mammalian cells (data not shown) and does not affect cellular growth. In order to study the localization of this protein to specific cellular structures, we fused the protein to the structural protein vimentin. The fusion protein exhibited a localization in the outer membrane that is characteristic of vimentin squiggles similar to live BHK-21 cells^40^. Well-structured vimentin in membranes is easily seen in images of in Hep2 cells (images taken with a Nikon Ti-E inverted microscope system with standard N-STORM setup, Fig. 10).

## Discussion

Previous studies have shown that tetramers of fluorescent proteins contain two different types of interfaces between interacting subunits: strongly charged (electrostatic AB) and hydrophobic (AC) interfaces. Based on the constructed spatial structure of SAASoti, we determined that lysines are present in the strongly charged AB interface. Chemical modification of the amino groups in SAASoti by succinic anhydride resulted in a transition from its aggregated into its monomeric form. Mass spectrometric analysis revealed that the succinylated form of the protein had several modified amino groups corresponding to K145, K149, and K172. Tetrameric SAASoti variants were obtained by single substitutions of K145 or K149 with isomorphic asparagine and glutamic acid residues, thus preventing SAASoti from higher levels of oligomerization. However, substitution of another lysine in the AB interface region (K172) did not result in altered oligomerization. Analysis of the spatial structure indicated that lysine 172 is juxtaposed to arginine 174 in the opposite polypeptide chain of the AB interface. This suggests that exchanging the ε-amino group for an amide group in asparagine can lead to the formation of a novel hydrogen bond between these residues.

Usually, site directed mutagenesis is used to introduce changes into both interfaces of a tetramer. In our case, monomerization was achieved by changing only one single position – V127T – in the hydrophobic AC interface of the tetramer interfaces. Thus, based on our results, we can hypothesize that in corals, SAASoti tetramers are assembled in two stages. First, dimers assemble by forming a hydrophobic AC interface between subunits, and subsequently two dimers interact to form a strongly charged AB interface. Thus, alterations in the hydrophobic AC interface result in the disruption of tetramer assembly.

**Figure 11:**
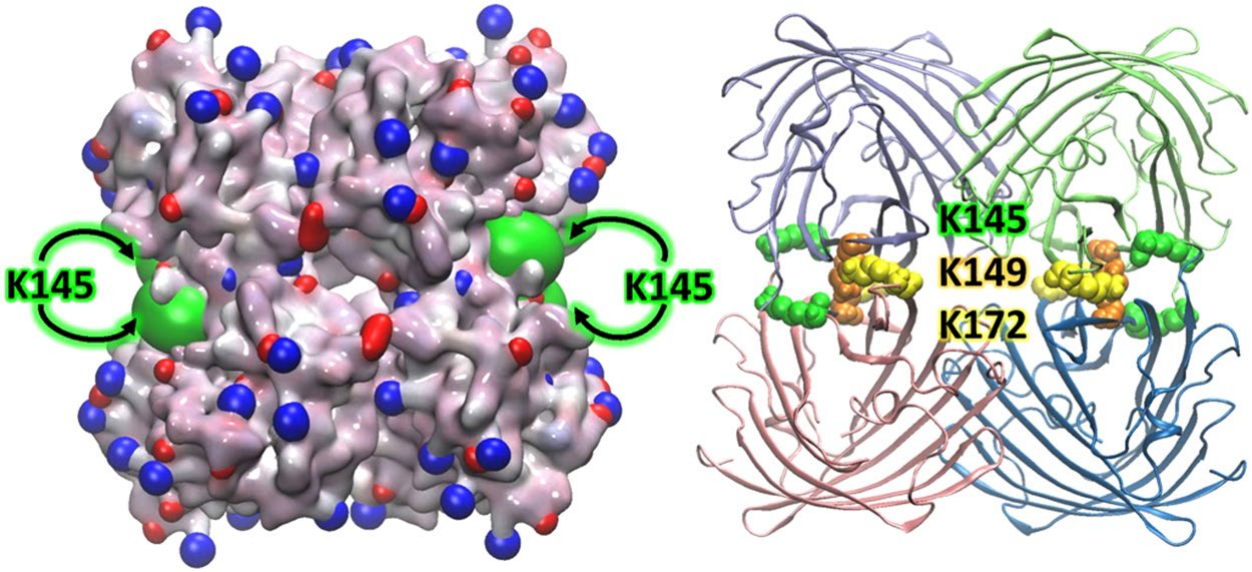
Charge distribution on the water-accessible surface (left) and the ribbon 3D model (right) of the SAASoti protein with highlighted lysine K145, K149 and K172. The model is based on the spatial structure of the Kaede protein. Negative (red) charges and positive charges (blue) are mostly paired at the surface of the tetramer structure. K145 lysine (green color in the left picture) is located in the cleft that is responsible for aggregation.

It is noteworthy that introduction of a single mutation led to the disruption of tetramer formation as well as formation of larger aggregates. It had been previously shown that aggregation of GFP-like proteins from corals critically depends on the positive charge of a protein’s N-terminus^11^. According to the surface charge distribution map of the SAASoti, an especially important area seems to be the cleft at the strongly charged (electrostatic AB) interface (Fig. 11). This suggests that the one tetramer with a (not yet identified) negatively charged area can interact with the positive charge at the K145 containing cleft of another tetramer. This may result in the formation of large aggregates of tetramers. Disruption of tetramer assembly would thus impair larger aggregate assembly.

The isomorphic amino acid residues that were chosen for site-directed mutagenesis did not alter the chromophore’s environment. All the mutant proteins retained their fluorescence.

The K145E and V127T mutants photoconvert from their green form to their red form much more rapidly than Dendra2 (Table 2). In the absence of specific localization signals, the V127T and K145E proteins are localized in the cytoplasm. The rapid photoconversion of the green form and the rapid photobleaching of the red form (Fig. 9A) may be highly useful for the protein’s application in sub-diffraction single-molecule localization microscopy in living cells. The high light intensities at 400 nm required by conventional FPs can cause significant photochemical damage to living cells^41^. The reduced light intensities required for photoconversion and photobleaching of V127T and other mutants could thus minimize the photochemical damage in sub-diffraction single-molecule localization microscopy.

**Table 2:** Comparison of the diffusion coefficients of SAASoti measured by 2fFCS.

	wt	K145E	V127T	eGFP
D(µm^2^/s) ± sd	72 ± 1	68 ± 1	93 ± 1	117 ± 2

**Table 3:** Fluorescent properties of SAASoti mutants and related photoconvertible proteins.

Protein name	Fluor. Form	Ex/Em, nm	Φ ± s.d.	ε, M^−1^cm^−1^ (a)	brightness
K145E SAASoti	Green	510/519	0.52 ± 0.05	66000	34320
	Red	573/579	0.34 ± 0.04	24000	8160
V127T SAASoti	green	510/519	0.59 ± 0.02	75000	44250
	Red	573/579	0.27 ± 0.03	24000	6480
Dendra2 (b)	green	490/507	0.50	45000	22500
	Red	553/573	0.55	35000	19250
mMaple (c)	green	489/505	0.74	15000	11100
	Red	566/583	0.56	30000	16800
mEos2 (c)	green	506/519	0.43	78000	33540
	Red	573/584	0.35	39000	13650

^a^Extinction coefficient at peak absorbance at pH 7.5, 10 mM Tris-HCl.

^b^Data from www.evrogen.com/products/Dendra2/Dendra2_Detailed_description.shtml.

^c^Data from McEvoy *et al*.^10^.

**Table 4:** Parameters for green and red forms of different SAASoti variants in cuvette and in live cells with standard deviation values^a^.

—	wt	K145E	K145N	K149E	K149N	K172N	V127T	Dendra2
**pK**_**1**_ (green form)	6.4 ± 0.1	6.0 ± 0.1	6.3 ± 0.1	5.7 ± 0.1	5.4 ± 0.1	4.0 ± 0.2	6.3 ± 0.1	6.6 ± 0.1
**pK**_**2**_ (red form)	6.7 ± 0.1	7.6 ± 0.1	7.6 ± 0.1	7.0 ± 0.1	7.1 ± 0.1	5.7 ± 0.1	6.6 ± 0.1	6.9 ± 0.1
**pK**_**1**_^**c**^ green-red photoconversion	6.4 ± 0.1	6.5 ± 0.1	6.3 ± 0.1	6.3 ± 0.1	5.8 ± 0.1	—	6.2 ± 0.1	6.9 ± 0.1
**pK**_**2**_^**c**^ red photobleaching at 400 nm	5.3 ± 0.2	6.3 ± 0.1	5.6 ± 0.1	5.7 ± 0.1	6.1 ± 0.1	—	6.5 ± 0.1	—
**k**_**1max**_, s^−1^ conversion	0.6 ± 0.1	0.72 ± 0.08	0.7 ± 0.2	0.9 ± 0.1	0.7 ± 0.2	—	0.7 ± 0.1	0.030 ± 0.001
**k**_**2max**_, s^−1^ bleaching at 400 nm	0.11 ± 0.06	0.11 ± 0.02	0.10 ± 0.02	0.09 ± 0.02	0.09 ± 0.03	—	0.08 ± 0.01	—
conversion **k**, s^−1^ HeLa cells conversion	—	—	—	—	—	—	0,33 ± 0.01	0,013 ± 0.002
bleaching **k**, s^−1^ HeLa cells at **560** nm	—	—	—	—	—	—	0,35 ± 0,1 0,042 ± 0,003	0,001 ± 0,001

^a^pK_1_, pK_2_ describe transition from anionic to neutral forms and are measured by fitting of spectral data at different pH according to formula (1) with Origin 8.5 software package (chapter «absorbance and fluorescent measurement»). pK_1_^C^ and pK_2_^C^ calculated by regression analysis of k_1_ and k_2_ obtained during photoconversion experiments according to formula (2) and (3) and Figs 8 and 9 (chapter “Photoconversion”). k_1max_ and k_2max_ obtained from formula (3).

## Methods

### Site-directed mutagenesis

SAASoti mutants (K145N, K145E, K149N, K149E, K172N, V127T, F104H, F104T and I147T) were generated by overlap extension PCR. Plasmid pET-SAASoti encoding the wild type protein, which was constructed earlier, was used as a template^30^. PCR was performed with high fidelity Kod Hot start DNA polymerase (Novagen, USA). The resulting fragments were gel-purified using QIAquick Gel Extraction Kit (QIAGEN, Germany), mixed and used in a second round of amplification to obtain full-length mutated versions of SAASoti. The resulting fragments were cloned into the NdeI and EcoRI sites of the expression vector pET22b (Novagen) to create pSAASoti K145N, pSAASoti K145E, pSAASoti K149N, pSAASoti K149E, pSAASoti K172N, pSAASoti V127T, pSAASoti F104H, pSAASoti F104T, and pSAASoti I147T. The presence of mutations was confirmed by DNA sequencing.

### Construction of plasmids for expression in mammalian cells

**Figure 12:**
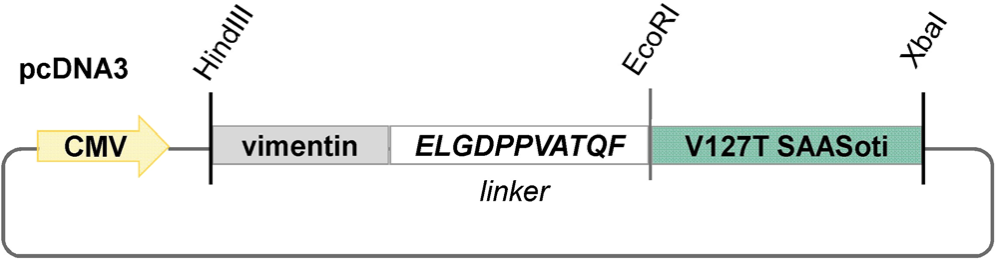
Scheme of V127T SAASoti fusion to the C-terminus of vimentin-gene via ELGDPPVATQF-linker cloned into pcDNA3 vector (Invitrogen).

The pcDNA3-V127T_SAASoti was constructed on the base of mammalian expression vector pcDNA3 (Invitrogen) and contains fused in frame Vimentin gene, linker sequence encoding ELGDPPVATQF, and V127T_SAASoti. Vimentin and V127T_SAASoti were amplified using pVimentin-Dendra2^42^ (kind gift from Dr. K. Lukyanov) and pSAASoti V127T as templates, and sequentially inserted into pcDNA3. Subsequently, linker sequence was introduced between Vimentin and V127T_SAASoti (Fig. 12).

### Protein expression and purification

Wild type and mutant proteins were over-expressed in *E. coli* BL21(DE3) as previously described^28^. Cells were harvested by centrifugation (5500 *g*, 15 min, 4**°**С) and resuspended in 40 ml of sonication buffer (20 mM Tris-HCl pH 7.5, 150 mM NaCl). Cells were disrupted using a french-press (Thermo Scientific). The obtained lysate was centrifuged for 15 minutes, 30000 *g*, 4**°**С. The colored supernatant, containing the protein of interest, was mixed with ammonium sulfate to 50% saturation and incubated for 4 hours at 4**°**С. A colored precipitate was collected by centrifugation at 3000 *g*, resuspended in 20 mM NaHCO_3_ and desalted on D-Salt columns (Pierce) in the same buffer. The protein was then purified using an ion-exchange MonoQ column (GE Healthcare) (equilibration buffer 20 mM NaHCO3, elution buffer 20 mM NaHCO_3_ 500 mM NaCl).

Protein purity was analyzed by gel-electrophoresis. SDS-PAGE was conducted in a Bio-Rad Mini Protean II system using hand-poured gradient (4–15%) polyacrylamide gels in a standard Tris-Gly buffer containing 0.1% SDS. Molecular weights were determined by using SM0431 Fermentas unstained protein markers. Gels were stained with PageBlue Thermo Scientific reagent (Fig. 2B).

### Chemical modification of protein

0.1 μM of SAASoti protein in 1 ml of 100 mM NaHCO_3_, containing 10% dimethylformamide (DMF), was mixed with a 10–20x molar excess of succinic anhydride in 100 μl of DMF and incubated overnight at 4 °С. Functional activity was controlled by measuring absorbance at 556 nm.

### Determination of oligomeric state by size exclusion chromatography

The size of protein complexes was determined using two methods: size-exclusion chromatography and FCS. Size-exclusion chromatography was performed using a Superdex 200 10/300 GL column, 20 mM Tris-HCl 150 mM NaCl pH 7.5 at elution rate of 0.5 ml/min using an AKTAPurifier 10 (GE Healthcare) with spectrophotometric detection. The column was calibrated using a Biorad Gel Filtration Standard, containing Thyroglobulin (bovine), γ-globulin (bovine), Ovalbumin (chicken), Myoglobin (equine), and Vitamin B12. Absorbance measurements were performed at 280 nm and 509 nm wavelengths.

### Two-Focus Fluorescence Correlation Spectroscopy (2fFCS)

The 2fFCS setup is based on a standard confocal epi-fluorescence microscope as described in detail in^36^. The system included water immersion Olympus objective (UPLAPO 60x W, 1.2 N.A.) two SPAD detectors (SPCM-AQR-14, Perkin Elmer), and two identical linearly polarized pulsed diode lasers at 485 nm wavelength (LDH-P-485, PicoQuant). We used 10 mM phosphate buffer, 150 mM NaCl pH7.4 and ~10^–9^ M proteins concentration at 25 °C. 2fFCS measurement time was 1 h for each measurement. Autocorrelation functions were fitted using a pure diffusion model for Atto488 (Supplementary Fig. S4(A)), FP’s (Supplementary Fig. S4(B,C)) was fitted by a triple state kinetics according to the method described in reference^36^. Atto 488 calibration gave an interfocal distance of 410 nm for the 2fFCS measurements.

### Mass spectrometry

After the protein treatment with succinic anhydride, which modified the available lysine residues, samples were purified using a reverse phase chromatography column (C18 P10). This was performed by adding 10 μl of sample to 10 μl of 0.1% trifluoroacetic acid (TFA). Washing was performed using 5 volumes (10 μl each) of 0.1% TFA, while elution was performed using 10 μl of 60% acetonitrile in 0.1% TFA. Samples were then dried and subjected to proteolysis with trypsin (Promega) in 0.05 M NH_4_HCO_3_ at a concentration of 10 μg/ml. Proteolysis was performed for 5 hours at +37°С, and stopped by addition of 20 μl of 0.5% TFA in a 10% solution of aqueous acetonitrile. This solution was used for mass spectrometry. 1 μl of sample solution and 0.5 μl of 2,5-dihydroxybenzoic acid (Aldrich, 20 mg/ml in 20% aqueous acetonitrile, 0.5% TFA) were mixed on a mass spectrometry target and air-dried. Mass spectra were obtained using a MALDI-TOF Ultraflextreme BRUKER mass spectrometer (Germany). Mass spectra were analyzed using FlexAnalysis 3.3 software (Bruker Daltonics, Germany). Comparison of experimentally determined and calculated masses of tryptic peptides of SAASoti was performed using Mascot software (www.matrixscience.com) Searches were performed by taking into account the possible modification of the ε-amino group of lysine and the N-terminal amino group with succinic anhydride, formation of a heterocycle in the fluorophore and the possible oxidation of methionine residues by atmospheric oxygen. In order to verify the presence or absence of modifications at specific ε-amino group of lysine residues modified by succinic anhydride we obtained fragmentation spectra for distinct peptides. Combined analysis of the MS + MS/MS results was performed with Biotools 3.2 (Bruker Daltonics, Germany) software.

### Absorbance and fluorescence measurements

Absorption spectra were measured in the range 250–700 nm using a Cary 300 (Varian, USA) spectrophotometer and a 3 mm quartz microcuvette. Samples were dissolved in 20 mM Tris-HCl, 150 mM NaCl, pH 7.5 buffer if not stated otherwise. A Cary Eclipse spectrofluorometer (Varian, USA) with a 3 mm quartz microcuvette was used for measurements for excitation in the range 250–600 nm and for fluorescence in the range 450–700 nm. Excitation and emission slits were set to 5 nm. Fluorescence spectra, photoconversion and photobleaching kinetics of the red form of the protein were obtained on a fiber optic SpectrClaster (Russia) spectrometer in the spectral range 400–900 nm, as described previously^43^ and on a Cary Eclipse fluorescence spectrometer (USA) in the spectral range 450–750 nm. Molar extinction coefficient ε of the green state was obtained as a 509/280 nm ratio (2.66) and 280 nm calculated absorbance multiplication^39^. Red state ε was determined in comparison to the green form. Quantum yields of green and red state for K145E variant of SAASoti were measured by comparison with green and red state of Dendra2 in the same 20 mM Tris-HCl 150 mM NaCl pH 7.5 buffer. To obtain pH-dependences we analyzed fluorescence intensity of proteins diluted by 1/20 into 200mM pH-adjusted buffers (sodium citrate ≤ 7.0, sodium phosphate ≥ 7.5). pK values were calculated using modified Henderson-Hasselbalch equation as described in^44^ according to equation 1:

1 $${H}^{+}+Chro{m}^{-}\mathop{\iff }\limits^{K}ChromH\,I([{H}^{+}])=\frac{{I}_{0}K}{K+[{H}^{+}]}$$

where anionic chromophore *Chrom*^−^ is fluorescent and ChromH isn’t. I – fluorescence intensity at current pH value, I_0_ – max fluorescence intensity at high pH values, K – equilibrium constant, [H^+^] – current equilibrium proton concentration. Data analysis was performed by fitting of experimental data according to formula (1) with Origin 8.5 software package.

### Photoconversion

Photoconversion was performed with Lumencor Spectra X LED (USA) illumination 30 mW at 395/25 nm in a 3 mm fluorescent microcuvette Hellma (Germany). Fluorescence spectra throw the conversion were obtained on a fiber optic SpectrClaster (Russia) spectrometer, as described previously^30^ and on a Cary Eclipse fluorescence spectrometer. Stock solutions of proteins were diluted 1/20 into 200mM pH-adjusted buffers (sodium citrate ≤7.0, sodium phosphate ≥7.0) to the final concentration ~1 μM.

Firstly, we calculated different rate constants in buffers with different pH-values. The fluorescence intensity as a function of time according to Fig. 9 was modeled with a bi-exponential function:

2 $${\rm{I}}({\rm{t}})={I}_{0}\cdot \frac{{k}_{1}}{{k}_{2}-{k}_{1}}\cdot (\exp (-{k}_{1}\cdot t)-\exp (-{k}_{2}\cdot t))+background$$

where I – fluorescence intensity at 590 nm in current time moment, I_0_ – max fluorescence intensity, *k*_1_ is the rate constant for red protein formation, and *k*_2_ is the photobleaching rate constant at 400 nm illumination for red form.

Secondly, we obtained dependences of rate constants against pH-values. Only protonated form of fluorescent protein (FPH) is photoconvertible whereas anionic (FP^−^) is not. Therefore, rate constants from Fig. 9 according to the reaction scheme from Fig. 8 are pH dependent and were analyzed by equation 3:

3 $${H}^{+}+F{P}^{-}\mathop{\iff }\limits^{{K}_{c}}FPH\,k=\frac{{k}_{max}[{H}^{+}]}{{K}_{c}+[{H}^{+}]}$$

where k is k_1_ or k_2_ (s^−1^) - current rate constants, k_max_ – max value of rate constants, K_c_ – equilibrium constant, [H^+^] – proton concentration. pK_1_^c^ and pK_2_^c^ were determined from pH profiles for k_1_ or k_2_ (Fig. 9) and values standard deviations weren’t exceed 0.2 value. All fittings were made by the non-linear regression analysis. Data analysis was performed with Origin 8.5 software package.

### Photoconversion in live cells

HeLa cells were transfected by pcDNA3/V127T-SAASoti and pcDNA3/Dendra2 using Torpedo Ibidi transfection reagent. Fluorescence was observed after 24 h. Fluorescent images were obtained using Olympus IX-71 with 100xTIRF objective. Lumencor SpectraX light engine with constant values of power light was used for photoconversion. We used 400 nm (0.2 mW before objective) light and 560 nm (1.3 mW) light for photoconversion and red-form excitation, respectively. Fluorescent cubes Olympus U-MWB (green form), U-MWG (red form) were used for image acquisition. Andor iXon Life 888 camera was used with following parameters: 1) SAASoti – 50 ms acquisition time, 500 – gain; 2) Dendra2 – 20 ms acquisition, 100 gain.

Image sequences were analyzed with ImageJ. Measured time kinetics of the red form were analyzed with a multiexponential decay model:

4 $$I=\sum {I}_{i}\cdot \exp (\,-\,{k}_{i}\cdot t)+c$$

where *I*_*i*_ is a pre-exponential factor: negative in case of growth and positive in case of bleaching; *k*_*i*_ (s^−1^) is a first order rate constant, and *c* is an offset.

### Nanocavity-based fluorescence quantum yield measurement

Fluorescence quantum yield of SAASoti V127T protein was measured using the absolute, calibration-free nanocavity-based method^45^. The core idea of the method is to measure the change of the fluorescence lifetime of a solution of fluorophores inside a planar metal nanocavity as a function of cavity length. The quantum yield can be defined as the ratio of the radiative (*k*_*rad*_) to the non-radiative (*k*_*nr*_) transition rate from the excited to the ground state of an emitter (Eq. 5):

5 $${\rm{\Phi }}=\frac{{k}_{rad}}{{k}_{rad}+{k}_{nr}}={k}_{rad}\tau ,$$

where *τ* is the excited state lifetime (fluorescence lifetime) of the fluorophore. Changing the cavity length modulates the local density of sates inside the cavity and thus the radiative transition rate *k*_*rad*_ of the enclosed fluorophores. By modelling the cavity-induced change of the fluorophores’ radiative rate k_rad_ and then fitting the model curve to the measured excited state lifetime τ modulation of the fluorophores inside the cavity, it is possible to determine an absolute value of its quantum yield. The measurement is based solely on the nanocavity-induced modulation of the radiative de-excitation rate of molecules and requires no comparison with fluorescence of a known sample.

**Figure 13:**
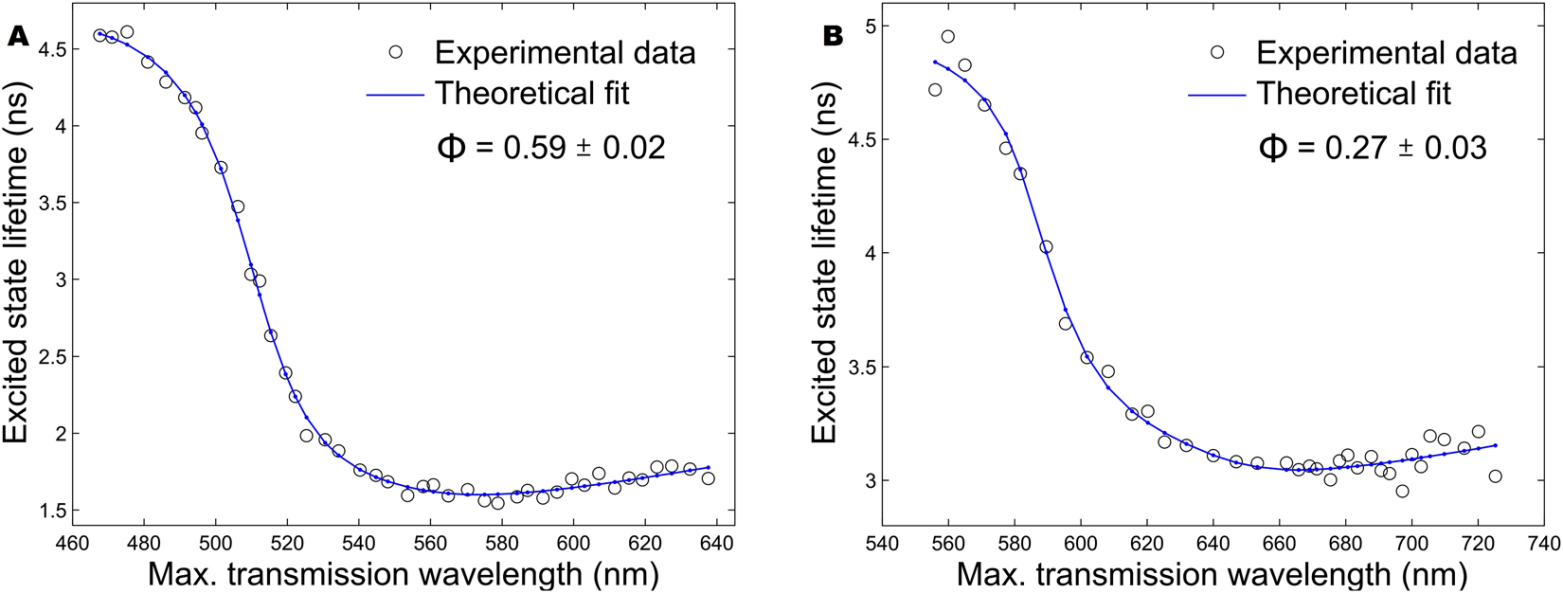
Nanocavity-modulated excited state lifetime of green (**A**) and red (**B**) forms of SAASoti V127T protein in 6 μM aqueous solution as a function of the maximum transmission wavelength of the cavity. Black circles are the measured data; blue curves are fits of the model. Φ indicates the values of the quantum yield.

Experimentally, we used a plasmonic nanocavity and a custom-built scanning confocal microscope as described in^37^. For measuring the quantum yield of proteins, we placed a droplet of 6 μM aqueous solution between the silver mirrors. The solid circles in Fig. 13(A,B) show the results of the fluorescence lifetime measurements at different maximum transmission wavelengths (linearly proportional to the cavity length) for green and red forms of SAASoti V127T protein, respectively. The solid curves are theoretical fits to the experimental data, which result in fluorescence quantum yield values of 59 ± 2% (green form) and 27 ± 3% (red form). The errors were estimated using a bootstrap algorithm.

The second free parameter in our theoretical model is the excited state lifetime of the fluorophore in the absence of the cavity. We used it to verify the validity of our quantum yield measurements by comparing the fitted lifetime values (i.e. extracted from the fit of our theoretical model of lifetime-versus-cavity-length dependence to the experimental data) with those that are directly measured in a droplet deposited on a pure glass cover slide. The discrepancy between the fitted and directly measured lifetime values did not exceed 5%, which indicates reliability of the obtained quantum yield values.

### PALM protocol

For PALM a Nikon Ti microscope (N-STORM system) with a SR Apo TIRF 100x objective was used. TIRF laser position of motorized TIRF illumination unit was set to about 4200 μ▯ and finely tuned to achieve highest contrast at the level of 1 μm above the coverslip surface^46^. For photoconversion/excitation 405 nm laser (5% intensity) and 561 nm laser (100% intensity) were used 43. Images were recorded with an Andor DU-897 camera operating in copped mode (256 × 256 pixels with 160 nm effective pixel size). Exposure time was 30 ms/frame. The system operated in sequential mode using excitation with a 405 nm laser for 1 frame and then excitation with a 561 nm laser for 3 frames (1 – registration of 561 nm signal, 2,3 – bleaching of the 561 nm signal), 500 cycles overall. Every second frame of a cycle was used for analysis. Images were analyzed using the ThunderSTORM plugin of ImageJ^47^.

### Preparation of Hep2 cells for imaging

pcDNA3/Vimentin-SAASoti-V127T, pcDNA3/Dendra2 and pcDNA3/SAASoti-V127T plasmids were expressed in *E. coli* DH5α strain and then isolated and purified using Endofree Plasmid Maxi Kit (Qiagen, Netherlands). The SAASoti V127T-vimentin cell line was obtained by liposomal transfection of Hep2 cells with plasmids using Torpedo reagent (ibidi, Germany). Protein expression is driven by strong CMV promoter. Cells were grown on Ibidi 35 mm dish in RPMI-1640 medium containing 10% fetal calf serum without antibiotics, for 4 days at 37 °C in a CO_2_ incubator. Fluorescence was measured with a Nikon Eclipse TE2000-U(Japan**)** system.

## Electronic supplementary material

Supplementary information
