# Supplementary material for: Monomerization of the photoconvertible fluorescent protein SAASoti by rational mutagenesis of single amino acids
Source: Sci Rep. 2018 Oct 19;8:15542. doi: 10.1038/s41598-018-33250-z (PMC6195611; doi:10.1038/s41598-018-33250-z)
Supplement: Supplementary file 1 — Supplementary information [file 41598_2018_33250_MOESM1_ESM.docx]

**­­Monomerization of the photoconvertible fluorescent protein SAASoti by rational mutagenesis of single amino acids**.

*Ilya D. Solovyev^1,2^, Alexandra V. Gavshina^1^, Aditya S. Katti^3^, Alexey I. Chizhik^3^*, *Leonid M. Vinokurov^4^*, *Grigory D. Lapshin^2^*, *Tatiana V. Ivashina^5^*, *Maria G. Khrenova^1,2^, Igor I. Kireev^6^, Ingo Gregor^3^,* *Jörg Enderlein^3^*, *Alexander P. Savitsky^1,2^**

^1^ A.N. Bach Institute of Biochemistry, Research Center of Biotechnology of the Russian Academy of Sciences, Moscow, Russia

^2^ M.V. Lomonosov Moscow State University, Department of Chemistry, Moscow, Russia

^3^ University of Göttingen, Third Institute of Physics – Biophysics, Friedrich-Hund-Platz 1, 37077 Göttingen, Germany

^4^ Branch of Shemyakin and Ovchinnikov Institute of Bioorganic Chemistry, Russian Academy of Sciences, Pushchino, Moscow Region, Russia

^5^ Skryabin Institute of Biochemistry and Physiology of Microorganisms, Russian Academy of Sciences, Pushchino, Moscow Region, Russia.

^6^ A.N. Belozersky Institute of Physico-Chemical Biology, M.V. Lomonosov Moscow State University, Moscow, Russia

* Author to whom correspondence should be addressed;

E-Mail: apsavitsky@inbi.ras.ru; Tel.: +7-495-954-65-12

**SUPPLEMENTARY FIGURES AND TABLES**

**Supplementary Table S1.** Calculated molecular weight (Mw) values for different FPs fractionated by size-exclusion chromatography on the Superdex 200 10/300 GL column (* – reducing conditions, 10 mM DTT; ** – after DTT removal) performed in 20 mM Tris-HCl pH 7.4, 150 mM NaCl at the elution rate of 0.5 ml/min using an AKTAPurifier 10 (GE Healthcare) with spectrophotometric detection (280 nm/ 509 nm).

| **FP** | **c, µM** | **V** | **Mw** |
| --- | --- | --- | --- |
| *eGFP* | 50 | 15.97 | **25.5** |
| *V127T* | 10 | 16.01 | **24.9** |
| *V127T* | 42 | 15.78 | **28.3** |
| *V127T* | 220 | 15.36 | **35.6** |
|  |  | 14.05 | **72.8** |
| ***V127T**** | **220** | **15.70** | **29.5** |
| *V127T *** | 100 | 15.81 | **27.8** |
| *V127T *** | 80 | 15.96 | **25.6** |
| *K145E* | 25 | 13.85 | **81.4** |
| *K145E ** | 25 | 13.86 | **81.0** |
| *WT* | 20 | 7.36 | **2858.5** |
|  |  | 11.3 | **329.5** |
|  |  | 12.52 | **168.8** |
|  |  | 14.22 | **66.5** |
| *WT** | 20 | 11.17 | **353.9** |
|  |  | 12.4 | **180.3** |
|  |  | 14.1 | **71.0** |
| *K172N* | 31 | 7.23 | **3069.7** |
| *K172N ** | 31 | 7.26 | **3019.6** |

Addition of reducing agent dithiothreitol (DTT) leads to disappearance of partially dimerized SAASoti fractions at the concentration of 0.22 mM. V127T SAASoti partial dimerization at high concentrations (0.22 mM) is most likely caused by S-S bond formation between the subunits. After DTT was removed from eluted fractions by dialyzing against 20 mM Tris-HCl pH 7.4, 150 mM NaCl and V127T SAASoti was concentrated we obtained 0.1 mM fraction. Repeated gel-filtration chromatography at non-reductive conditions revealed monomeric state of the protein. Structure analysis of SAASoti model revealed two surface-facing cysteine residues – C21 and C117. Interestingly, that wild type SAASoti still forms stable oligomers under reductive conditions, as only 7.36 ml peak disappears. DTT addition in the case of K145E and K172N SAASoti variants, existing as oligomers in solution even at the concentrations about 30 μM, does not lead to the protein monomerization. Thus, one can conclude that V127T mutation disturbs strong a.a. interactions of another chemical nature between the subunits in dimers and tetramers.

**Supplementary Figure S1.** MALDI-TOF/TOF MS Spectra of wild type (A) and succinylated wild type (B) SAASoti.
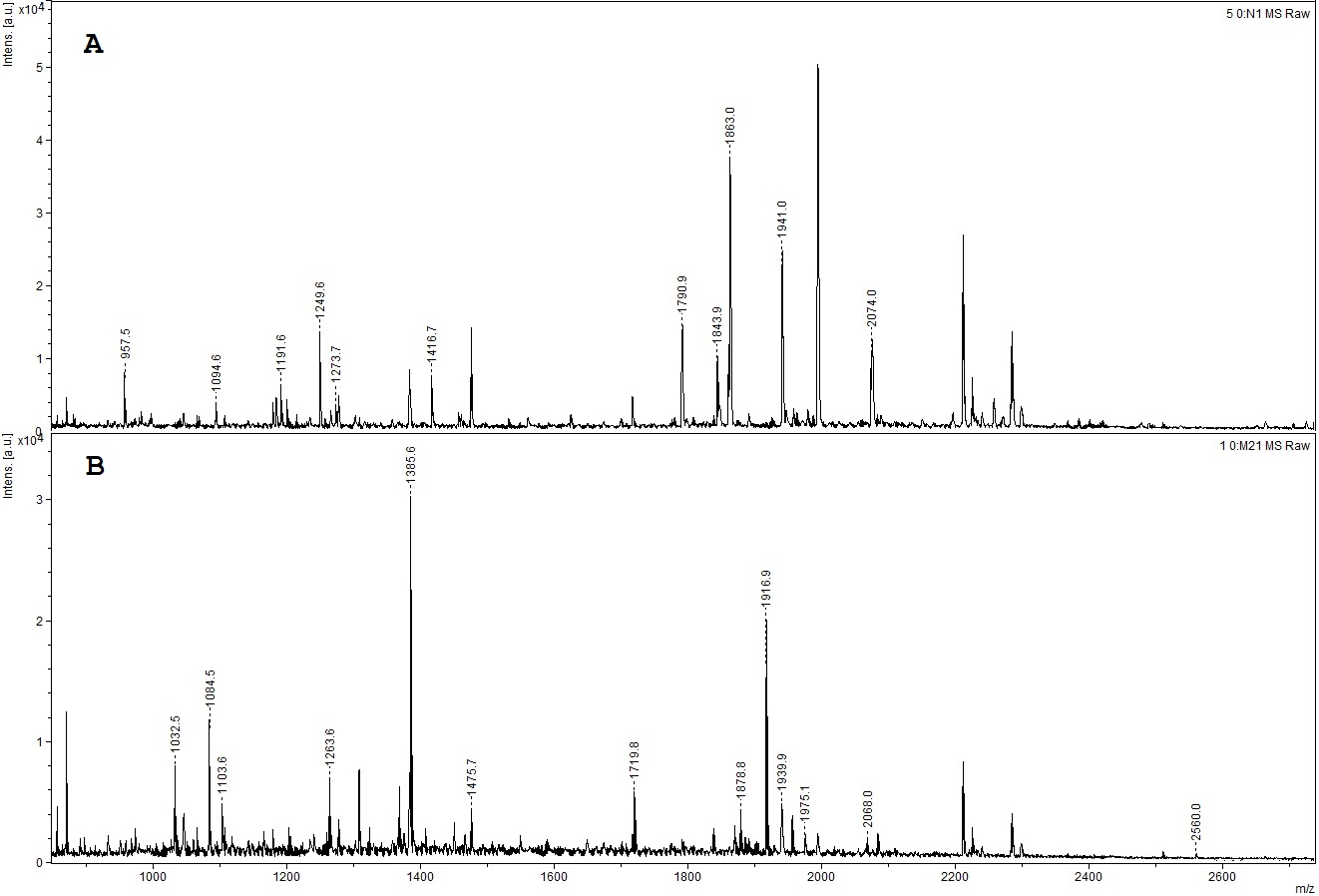


**Supplementary Figure S2.** MALDI-TOF/TOF MS/MS spectrum of the molecular ion at
m/z = 1032.5. K + 100,7 Da corresponds to K139 succinylation in -KTIQWEK- tryptic peptide.

**Supplementary Figure S3.** Peptides identified using MALDI TOF/TOF are highlighted in grey. Succinylated lysines are highlighted in red. Non-modified residues are highlighted in blue (not seen in the peptides) and green . Uncovered lysines are yellow.

1 10 20 30 40 50 60

MALSKQYIPD DMELIFHMDG CVNGHYFTIV ATGKAKPYEG KQNLKATVTK GAPLPFSTDI

70 80 90 100 110 120

LSTVMHYGNR CIVHYPPGIP DYFKQSFPEG YSWERTFAFE DGGFCTVSAD IKLKDNCFIH

130 140 150 160 170 180

TSMFHGVNFP ADGPVMQRKT IQWEKSIEKM TVSDGIVKGD ITMFLLLEGG GKYRCQFHTS

190 200 210 220

YKAKKVVEMP QSHYVEHSIE RTNDDGTQFE LNEHAVARLN EI

In spite of the fact that only 47% of the entire sequence is found, 12 of the 17 lysines are located in the tryptic peptides. Moreover, as trypsin cleaves peptide bond at the carboxyl side of the lysine residues K34, K84 and K184 (corresponding tryptic peptides: 1275.7, 1385.6 and 2068.0, Table 1), these residues were not exposed to succinyl modification. In other words, 15 of the 17 lysine residues were identified as modified or not (88% lysine coverage).

**Supplementary Figure S4.** Size-exclusion chromatography of 0.22 mM V127T SAASoti. Superdex G200 100/20 GL column, detection by absorption at 509 nm. Black line: 20 mM Tris-HCl pH 7.4, 150 mM NaCl; red line (reducing conditions) 20 mM Tris-HCl pH 7.4, 150 mM NaCl, 10 mM DTT.


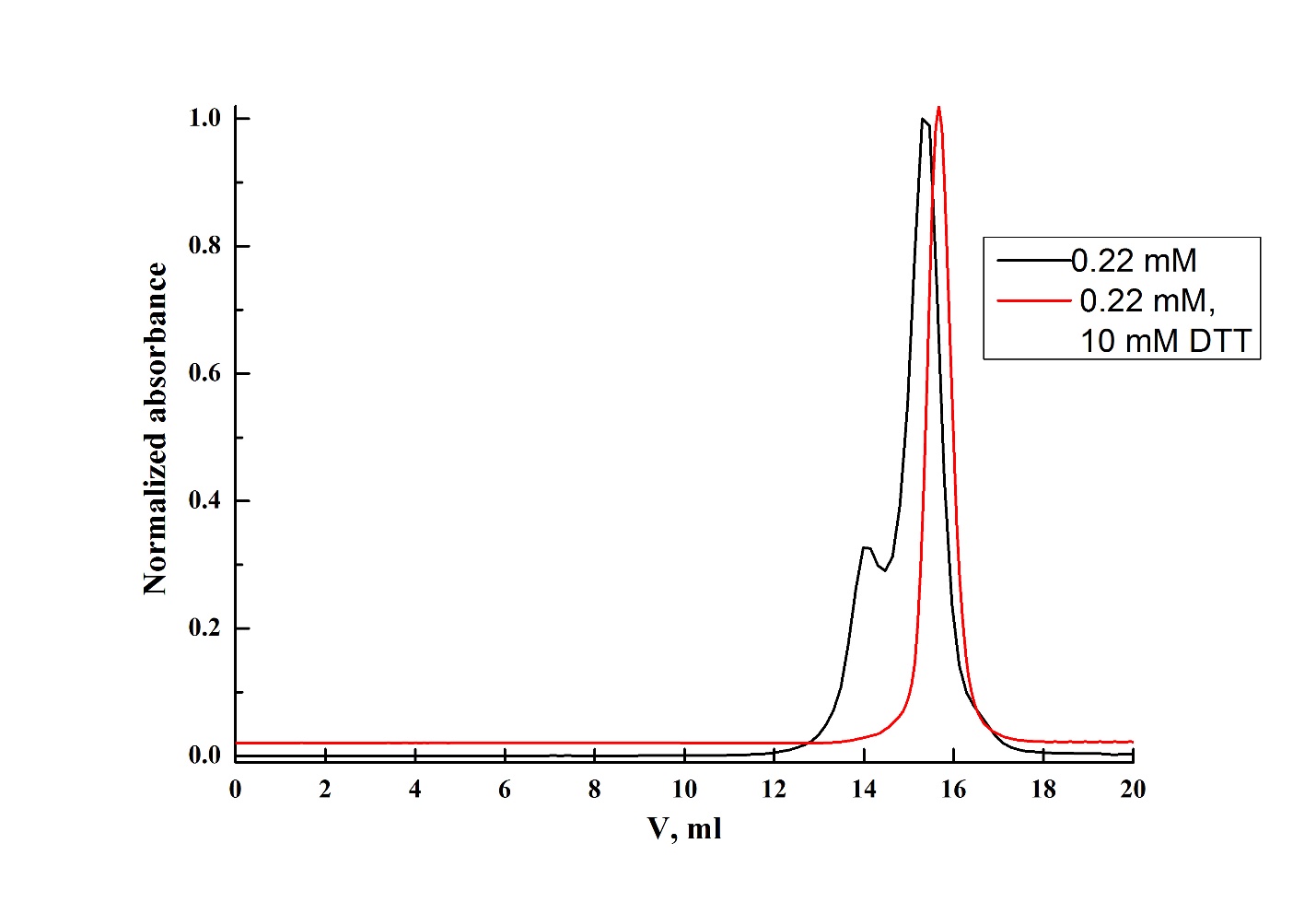


**Supplementary Figure S5.** 2fFCS measurements of fluorescent proteins (FP) diffusion. Callibration curves were fitted by a pure diffusion model, FPs’ curves were fitted by a triplet model, τ – triplet time. Dots – data points, lines – fitting curves. Green and cyan curves – first and second focus autocorrelation functions, red and blue curves – cross-correlation curves between two focal volumes. A – Atto488 calibration, B – eGFP, C – wild type SAASoti.

| 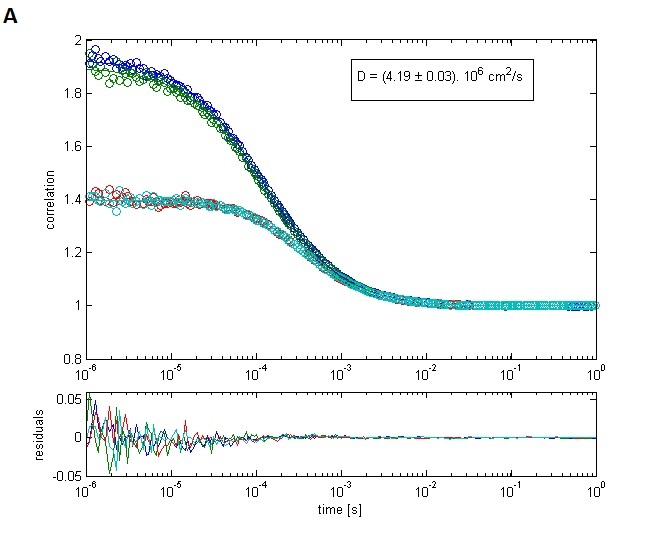 |
| --- |
| 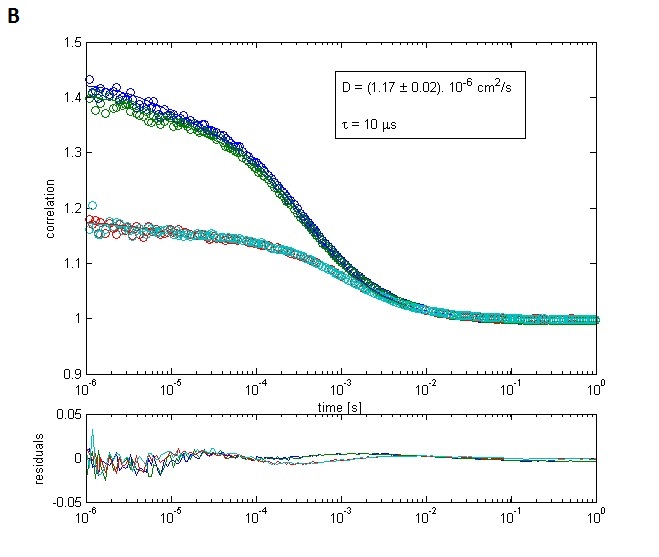 |
| 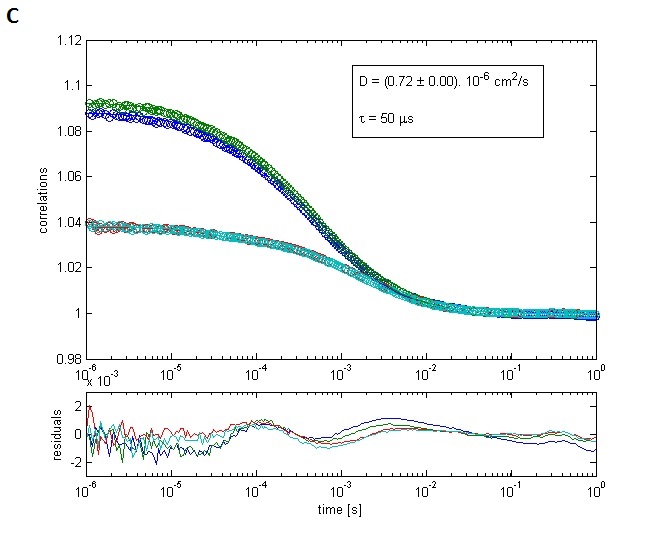 |

The 2fFCS setup is based on a standard confocal epi-fluorescence microscope as described in detail in ref. [1]. Two identical, linearly polarized pulsed diode lasers at 485 nm wavelength (LDH-P-485, PicoQuant, Berlin, Germany) are combined by a polarizing beam splitter (Narrow Band Polarizing Beamsplitter Cube 488, Ealing Catalogue,St. Asaph, UK). The lasers are pulsed alternately with an overall repetition rate of 40 MHz and have a pulse duration of 50 ps each. Alternate pulsing is accomplished by special laser driver electronics (PDL 808 “Sepia”, PicoQuant, Berlin, Germany). Both beams are then coupled into a polarization maintaining single mode fiber with a collimated output after. This is the pulsed interleaved excitation (PIE) [2] mode with alternating orthogonal polarization. The beam is then reflected by a dichroic mirror towards the microscope’s water immersion objective (UPLAPO 60x W, 1.2 N.A., Olympus Europa, Hamburg, Germany). Before entering the objective, the light beam is passed through a Nomarski prism (U-DICTHC, Olympus Europa, Hamburg, Germany) that is normally exploited for differential interference contrast (DIC) microscopy. The principal axes of the Nomarski prism are aligned with the orthogonal polarizations of the laser pulses, so that the prism deflects the laser pulses into two different directions according to their corresponding polarization. After focusing the light through the objective, two overlapping excitation foci are generated, with a small lateral shift between them. The distance between the beams is uniquely defined by the chosen DIC prism and is in our system equal to 410 nm (see Results). Fluorescence is collected by the same objective (epi-fluorescence setup), passed through the DIC prism and the dichroic mirror, and focused into a single circular aperture (diameter 150 µm) which is positioned symmetrically with respect to both focus positions and chosen large enough to let the light pass from both foci. After the pinhole, the light is collimated, split by a non-polarizing beam splitter cube (Linos Photonics GmbH & Co. KG, Goettingen, Germany), and focused onto two single-photon avalanche diodes (SPADs, SPCM-AQR-14, Perkin–Elmer, Wellesley, MA, USA). 500 nm long pass and 525/45 nm emission filters (Semrock, USA) were used. Photon correlation was calculated only between photons of different SPADs for avoiding any of the SPAD afterpulsing, see for example, ref. [3]. A dedicated single-photon counting electronics (PicoHarp 200, PicoQuant, Berlin, Germany) is used to record the detected photons. The electronics operates in time-tagged time-resolved (TTTR) mode,[1] recording for every detected photon its macroscopic arrival time with 100 ns temporal resolution, and its arrival time with respect to the last laser pulse with picosecond temporal resolution (time-correlated single photon counting, TCSPC[4]). Autocorrelations as well as cross correlation functions between the two detection volumes and fits were calculated by custom-written software on a PC using MATLAB.

[1] M. Bohmer, F. Pampaloni, M. Wahl, H. J. Rahn, R. Erdmann, J. Enderlein, Rev. Sci. Instrum. 2001, 72, 4145 – 4152.

[2] B. K. Muller, E. Zaychikov, C. Brauchle, D. Lamb, Biophys. J. 2005, 89, 3508 –3522.

[3] J. Enderlein, I. Gregor, Rev. Sci. Instrum. 2005, 76, 033 102

[4] D. V. O’Connor, D. Phillips, Time-Correlated Single Photon Counting, Academic Press, London, 1984.
